# Improving the quality of Peer Review and accelerating the peer review process

**DOI:** 10.12669/pjms.39.1.7236

**Published:** 2023

**Authors:** Shaukat Ali Jawaid

Finding good quality of peer reviewers is one of the most important problems faced by the editors of biomedical journals in developing, Low and Middle Income countries.^1^ It is one of the reasons for delay in processing and publication of manuscripts submitted to the journals but authors are very impatient to get feedback and wish to get their work published as soon as possible after submission putting lot of pressure on editors.^2^ At the same time it is also the duty and responsibility of the editors that they should ensure speedy processing, give the authors feedback and do not sit on the submissions for weeks and months and then reject the papers. Authors spend lot of time, resources on conducting the study, preparing the manuscripts and are keen on their publication to meet some academic requirement. Hence, it becomes essential that the editors should accelerate the peer review process, increase their reviewer’s database, go for innovations and train new reviewers.

It was with this background that a few months ago we started an experiment to ask the authors to suggest two to three potential reviewers for their studies. This is also being practiced by many other journals. There is nothing wrong with this. Recently issued guidelines by Committee on Publication Ethics (COPE), Directory of Open Access Journals (DOAJ), and Open Access Scholarly Publishing Association,(OASPA) has also included the use of author recommended reviewers.^3^ However, it is made clear to the authors that we might use one of them along with reviewers from our database hoping that it may solve the problem of reviewer’s scarcity to some extent and also help us increase reviewers in our database. The authors are also informed that comments from the suggested reviewers are subject to their acceptance. In case of submissions from overseas, what we do is that once the authors suggest reviewers, we first check their credentials, look at their institution affiliation, are they in the faculty, check and recheck, confirm their e mail, look at their publications on the net, what are their academic accomplishments and once we are convinced with their academic interest, we send them a manuscript for review on trial basis. They are also sent guidelines on how to review a manuscript and how to write peer review report. Peer Review Performa sent as an attachment also contains about two dozen questions for review of an original study which helps them in reviewing the paper. The reviewers are also supposed to point out what new information this study has added to the medical literature, what is its significance, relevance in clinical practice apart from looking at numerous other aspects of the study. Different Reviewers Proforma are used for Reviews, Editorials and Case reports.

If we think the report is vague or substandard, our observations are conveyed to the suggested reviewer as well as the authors who had proposed their name. The review is discarded and the manuscript is sent to another reviewer. We have found it quite useful. Sometime the review is very comprehensive, hence those reviewers are asked to share their CV and they are included in our reviewer’s database. Those not found good enough, are never asked to review again. As with some other reviewers, they are offered some incentives, their own submissions are processed on priority and they also get little preference in publication.

In case of submissions from within the country, the authors are advised not to suggest reviewers from their own institution and from the same city. These initiatives has been of some help in accelerating the peer review process.

Academicians in our reviewers database, enjoy certain privilege and every effort is made to retain them and benefit from their expertize. Some of the privileges we offer are as under:

1. In case they submit their manuscripts for publication in the journal, the processing charges are waived.
2. They get some priority in processing of their manuscript.
3. They get discount in publication charges once their paper is accepted for publication after peer review and this ranges between five to eight thousand rupees.
4. They also get some discount in publication if they are one of the listed authors in the manuscript.
5. They can also recommend some discount in publication charges of their trainees.
6. However, there are others who do not submit their own papers for publication. Hence, they are sent some gifts of books from time to time, they are facilitated to join some training workshop or participate in a conference by sponsoring their registration.

Since all these suggested reviewers are also provided with guidelines on how to review a paper, how to write peer review report, appropriate peer review Performa is shared with them, it helps in training these reviewers as well.

Every journal is also supposed to frequently organize workshops on scientific writing and publishing, training of peer reviewers from time to time. Since it is the responsibility of the editor to maintain standard of the journal, ensure that quality of manuscripts being published is of high quality which warrants that quality of the peer review is outstanding and exceptional. Good quality manuscripts ensures greater citations thus further improving the Impact Factor of the journal. It also depends a lot on the editor as to how much weightage they give to the reviewers comments who work as advisors to the Editor who has the final authority to accept or reject a paper.^4^,^5^ Those manuscripts which also contain and cite manuscripts published in the journal in which they wish to make the submission, helps in a positive decision by the Editor to accept the paper for further processing. It is up to the editor whether to accept the suggested reviewers or not, and in case of a positive decision, the peer review comments are carefully reviewed before they are communicated to the authors.

## Pre submission review of abstract:

Since processing fee is non-refundable, we ask authors to share with us the structured abstract of their proposed submission. It must contain all the details i.e. complete details of listed authors, their institutional affiliation and individual author’s contribution. Incomplete abstracts without list of authors and their affiliation are not entertained. Those found interesting, informative and have some innovation are then asked to make the submission on the journal website with all the documents i.e. Ethics Committee approval, Letter of Undertaking signed by all the authors, scanned copy of the processing fee paid. Incomplete submissions are not entertained. This saves lot of inconvenience to the authors and in case their submission cannot be processed further, the decision is conveyed to them within a few days and they also save the processing fee. We are also considering for some “Token appreciation” to those reviewers who do not avail other benefits.

## Pre submission review of paper:

Some journals also offer pre submission review of papers before authors are asked to submit on the journal website. Since they have lot of editorial staff, they can manage to offer this facility but it is not possible for journals with meagre editorial staff. Of course the authors have to pay for this service as well. It has the advantage that if the paper is not accepted for further processing, the authors can submit it to some other journal without wasting time.

## Fast Track Processing of manuscripts:

We used to offer this facility in a few selected cases when the authors had to sit in a Fellowship Exam by CPSP, or complete their PhD Thesis requirements but had to discontinue this soon after. Every author wished to avail this fast track processing facility and this would have meant that those with financial resources will get their papers published much earlier than others. Hence, we were forced to discontinue this facility as due to human resource constraints, it is not possible to process too many papers every month. Since most of the authors are not fully aware of the whole submission, processing and publication process, they fail to appreciate all this and insist on fast track processing. Editors cannot please everyone, when they refuse such requests, the authors get annoyed. 6 The solution lies in having more and more good quality standard peer reviewed biomedical journals published from Pakistan. It will not only reduce the pressure on the only three Impact Factor Journals published from Pakistan but also help authors in early publication of their research work.

Keeping this objective in mind, Pakistan Association of Medical Editors (PAME) in collaboration with University of Health Sciences (UHS) started a six months course in Medical Editing (CME) in 2019. So far four batches have qualified and one hundred twenty five healthcare professionals have qualified. It helped us in achieving our objective as most of the biomedical journals which have acquired the services of these CME qualified healthcare professionals, their journals have been recognized by the Higher Education Commission. An Advance course which is also of six months duration which is proposed to be named as Diploma in Medical Journalism has also been started and the first batch has completed its training.^7^ In this the second contact session included visit to all the three Impact Factor Journals in Pakistan at Karachi so that they can see and observe their working as all the three have different business models. Feedback shows that they learnt a lot and found it very useful.

Higher Education Commission of Pakistan has so far recognized sixty eight biomedical journals. It includes three journals i.e. Paksitan Journal of Medical Sciences, Journal of College of Physicians & Surgeons Pakistan and Journal of Pakistan Medical Association which are placed in “X “category. These journals also enjoy Impact Factor. The rest sixty five are in Y Category.^8^ A few medical journals published by some medical institutions have also been included in JCR by Clarivate and it is hoped they will also get an Impact Factor in the coming days. We need to improve the quality and standard of other medical journals still in “Y” category so that they can also move to the next category. All this will help the researchers to publish their research work in these journals, they won’t have to wait for too long as publication in journals overseas is very expensive and many cannot afford it.

Yet another option could be that if the regulatory authorities in Pakistan do not insist on having a print copy as well by the medical journals, at least from those which enjoy Impact Factor and are being published regularly for the last fifteen to twenty years, are covered by important databases like Scopus, these journals will be able to accommodate more papers. Online only journals offer lot of benefits and when the whole world is going for digitalization, one wonders why the regulatory bodies in Pakistan cannot have a rationale approach to help researchers and journal editors.^9^ This issue has been brought to the notice of regulatory bodies time and again but so far there has been no positive developments. Libraries have no place to keep record of these print copies of the journals while the journals themselves are faced with a dilemma how to maintain record of old issues for years. The regulatory bodies will have to take some decision sooner or later or the Journals may be forced to take a decision on their own to do away with the print copies and just be content with online publications. In view of the ever increasing cost of production, this appears to be only viable option. We in Pakistan Journal of Medical Sciences are seriously considering to opt for Online Journal only and do away with the print version next year. Whenever such a decision is taken, authors will be informed well in advance.
